# Locally Adjust Networks Based on Connectivity and Semantic Similarities for Disease Module Detection

**DOI:** 10.3389/fgene.2021.726596

**Published:** 2021-10-25

**Authors:** Jia Liu, Huole Zhu, Jianfeng Qiu

**Affiliations:** ^1^ State Key Laboratory of Media Convergence and Communication, Communication University of China, Beijing, China; ^2^ Key Laboratory of Intelligent Computing and Signal Processing of Ministry of Education, School of Artificial Intelligence, Anhui University, Hefei, China; ^3^ Information Materials and Intelligent Sensing Laboratory of Anhui Province, School of Artificial Intelligence, Anhui University, Hefei, China

**Keywords:** complex disease, module identification, protein-protein interaction network, locally adjust networks, connectivity and semantic similarities

## Abstract

For studying the pathogenesis of complex diseases, it is important to identify the disease modules in the system level. Since the protein-protein interaction (PPI) networks contain a number of incomplete and incorrect interactome, most existing methods often lead to many disease proteins isolating from disease modules. In this paper, we propose an effective disease module identification method IDMCSS, where the used human PPI networks are obtained by adding some potential missing interactions from existing PPI networks, as well as removing some potential incorrect interactions. In IDMCSS, a network adjustment strategy is developed to add or remove links around disease proteins based on both topological and semantic information. Next, neighboring proteins of disease proteins are prioritized according to a suggested similarity between each of them and disease proteins, and the protein with the largest similarity with disease proteins is added into a candidate disease protein set one by one. The stopping criterion is set to the boundary of the disease proteins. Finally, the connected subnetwork having the largest number of disease proteins is selected as a disease module. Experimental results on asthma demonstrate the effectiveness of the method in comparison to existing algorithms for disease module identification. It is also shown that the proposed IDMCSS can obtain the disease modules having crucial biological processes of asthma and 12 targets for drug intervention can be predicted.

## 1 Introduction

There exist a number of complex diseases, which are not caused by the malfunction of an individual gene product, but the dysfunction of biological systems formed by several disease-related genes (Zheng et al., 2006; Zheng et al., 2008; Schadt, 2009; Zanzoni et al., 2009; Albert-László et al., 2011; Su et al. 2019). These disease-related genes and their products (e.g., proteins) are not randomly distributed on a molecular network, but they prefer to work together as a group for similar biological functions Sol et al., 2010. The above evidence suggests the existence of disease modules, which were firstly defined by Barabasi et al. as the connected subgraphs formed by proteins associated with a disease (Menche et al., 2015). The disease modules can be considered as the characteristic of a particular disease phenotype (Susan Dina et al., 2015). It becomes quite important to identify the disease modules, which is helpful for understanding the molecular mechanisms of disease origin and progression, and thus aiding the identification of synergistic drug combinations (Cheng et al., 2019).

With the rapid accumulation of protein-protein interactions, the investigation of interactions between proteins in the human protein-protein interaction (PPI) networks has become one of the primary approaches for detecting disease modules of complex diseases (Igor et al., 2008; Sebastian et al., 2008; Wang et al., 2011). These approaches usually are performed by using the connectivity information in the PPI network, and can be roughly classified into four categories, i.e., neighborhood scoring methods (Krauthammer et al., 2004; Jonsson and Bates, 2006; Tu et al., 2006; Xu and Li, 2006), seed expanding-based methods (Sharma et al., 2012; Susan Dina et al., 2015; Zhang et al., 2017b), diffusion-based methods Sebastian et al. (2008) and representation learning methods (Härtner et al., 2018). However, the disease modules achieved by these connectivity-based approaches usually show insufficient reliability to illustrate a specific disease phenotype, since nearly 80*%* of actual associations between proteins are not included in the existing PPI network and these missing associations leave many disease proteins isolated from their disease modules (Menche et al., 2015). Besides, high throughput experiments often produce a large number of interactions with noise, which makes several irrelevant proteins included in the disease module (Cho and Montanez, 2013).

To obtain better detection results, several studies have been performed by combining the protein-protein interaction data with other types of biological data, such as sequence-based features, epigenomic data, gene ontology (GO) annotation and expression patterns (Csaba and Mauno, 2009; Franke et al., 2006b; Liu et al., 2015). Among these biological data, GO annotation has shown to be an effective semantic resource which usually serves as a complement to protein-protein interactions to reflect functional information, where the semantic information of a gene is defined as the molecular function of genes and the biological processes in which the genes are involved (Franke et al., 2006b; Liu et al., 2015). Disease modules achieved by existing approaches have shown the ability to combine the connectivity information with the semantic information for the prioritizing of candidate disease genes (Franke et al., 2006b; Liu et al., 2015). For example, in Franke et al. (2006b), a gene network is developed by the intergation of the GO annotation information, interactions between proteins and microarray coexpressions, and genes are ranked based on the network. In Liu et al. (2015), Liu et al. proposed a method combining the topological similarity in the PPI network with the semantic similarity to select the candidate disease genes. However, the detection results of existing methods need to be further improved, since several unreliable interactions will hinder the detection effectiveness.

Recent studies on complex networks show that an ambiguous community structure can be converted into a structure much clearer than the original one by adding and reducing several links in the network (Su et al., 2021). It is known that about 80*%* of the disease proteins are disconnected from disease modules because of the incomplete biological network, where these proteins tend to be localized in the neighborhood of the disease modules (Menche et al., 2015). This means that the implementing of removing associations from the PPI network and adding into associations around the known disease proteins can compensate for the incomplete and incorrect interactions between the proteins in the PPI network, which will facilitate the detection of disease modules. For this reason, we proposed a connectivity and semantic similarities based method (termed as IDMCSS) to identify disease modules by locally adjusting a given PPI network in the detection process in a conference paper (Su et al., 2020). The connectivity similarity reflects the closeness of proteins based on protein-protein interactions and the semantic similarity represents functional similarities of proteins based on GO annotation information. In Su et al. (2020), due to the page limitation, the IDMCSS was only briefly presented and some simple experiments demonstrated the effectiveness of the algorithm for disease module identification. In this paper, we give an extended version of the paper in Su et al. (2020) by adding more analysis and discussions on the algorithm. Specifically, we present a detailed description of the strategies used in the IDMCSS and a series of experimental results are reported with detailed discussions to illustrate the competitiveness of the IDMCSS. We also add the related work section to highlight the difference between the IDMCSS and existing algorithms, as well as the complexity analysis of the IDMCSS. To sum up, the IDMCSS algorithm contains the following two main contributions:

1) A strategy of network structure adjustment is proposed to locally change the structure of the existing PPI network by adding several missing links which are likely to be related to disease proteins and removing some existing links which have an extremely weak correlation to disease proteins. To this end, the strong-linked or weak-linked proteins are firstly selected from the neighbors of disease proteins, where the strong-linked proteins and the weak-linked proteins have large and small connective similarities with disease proteins, respectively. Then, two key operators, i.e., adding link operator and removing link operator, are designed to add several links between strong-linked proteins and disease proteins, and remove some links between strong-linked proteins and disease proteins.
2) A disease module detection method IDMCSS is proposed by using the strategy of network structure adjustment based on both connective and semantic similarity. In the proposed method, a strategy to expand the set of disease proteins is tailored for the disease module identification. The proposed IDMCSS is verified to be superior over some representative disease module identification approaches.

The rest of the paper is organized as follows. Section 2 presents the disease module detection problem and reviews the related methods for disease module identification. Then, we describe the details of the proposed algorithm in Section 3. Section 4 shows the experimental results and Section 5 concludes the paper and gives the future work.

## 2 Related Work

Recently, the PPI network has become a popular resource for disease module identification (Cagney et al., 2000; Navlakha and Kingsford, 2010). Several disease protein prioritization strategies have been developed to detect disease modules by taking advantage of the existing PPI networks (Agrawal et al., 2017; Cui et al., 2018; Tian et al., 2020). Due to the unreliability of the connective information, there exist some disease modules that are not observable in the PPI networks (Wu et al., 2013). There are also some approaches which are performed by combining connective information and other information such as GO annotation information and expression patterns, to change the structure of the PPI networks (Liu et al., 2015; Franke et al., 2006a; Luo and Liang, 2015; Zhang et al., 2017a). In what follows, we only recall several approaches based on changing network structure, which can be roughly divided into two groups.

The first group changes the network structure by adding several potential missing links to make the network more reliable or adding extra nodes to connect disassociated disease proteins. In order to achieve a reliable network, Franke et al. (2006a) collected a set of validated protein-protein interactions and made use of GO annotation, coexpression data to predict interactions of the remaining protein pairs by a Bayesian classifier. The achieved network was applied to detect candidate disease proteins. To avoid spurious interactions in the PPI networks, a network was reconstructed by connecting pairs of disconnected proteins in the PPI network whose higher-order topological similarities were larger than a certain threshold, where the higher-order topological similarity between two proteins was measured by a link prediction algorithm. Then, candidate inherited disease proteins were prioritized by a random walk-based algorithm on the reconstructed network (Luo and Liang, 2015). Based on a similar idea, Liu et al. developed an algorithm (CTSS) to detect disease proteins by adding the weak interactions between genes which were not connected in the existing network based on the semantic similarity between them (Liu et al., 2015). Experimental results indicated that the PPI network became more perfect by involving reliable associations. In order to connect known disease proteins to be a coherent network module, a seed connector algorithm was developed to detect disease modules by adding as few extra hidden proteins to the set of known proteins as possible (Wang and Loscalzo, 2018). The newly added proteins have been demonstrated useful, since they show significant biological relevance in terms of their functional similarity to known disease proteins and their enrichment of drug targets.

The second group focuses on eliminating potential incorrect associations in the existing networks to achieve a more reliable network or removing several links which are not related to a particular disease phenotype to obtain a disease-specific network. For instance, in order to eliminate potential incorrect associations, the structure of the human PPI network is adjusted by measuring the correlation coefficient between a pair of connected proteins and removing those with a low correlation coefficient (<0.75) in gene expression data (Liu et al., 2011). In Zhang et al., 2017a), a gene co-expression network was constructed according to the expression patterns of genes, and the links which were not included in the gene co-expression network were removed from the existing PPI network to improve the prediction accuracy of disease proteins. As for a disease-specific network, only the interactions between the immunome proteins in the PPI network were taken into account for the construction of primary immunodeficiencies network, where no new nodes were added, and proteins without interactions were removed (Ortutay and Vihinen, 2008. Similarly, in Bragina et al. (2016), an associative network, which represents molecular interactions between proteins and genes associated with Tuberculosis, was reconstructed and analyzed, and new candidate genes for TB susceptibility were discovered.

Although various network structure based techniques have been developed for the identification of disease modules, traditional approaches are still far from satisfactory, since little approaches focus on dealing with the missing and incorrect links simultaneously. In this paper, we propose a disease module identification method, which is achieved by both adding several potential missing interactions and removing several potential incorrect interactions from the existing PPI networks, based on two types of data, i.e., connective information and semantic information of proteins.

## 3 The IDMCSS Method

In this section, we give the details of the proposed IDMCSS algorithm. Firstly, the general framework of IDMCSS is presented, and then the network adjustment strategy as well as the way to identify disease proteins which are the main components of IDMCSS are elaborated.

### 3.1 Framework of IDMCSS

The proposed IDMCSS is a network-based disease module detection method, where the keypoint is to expand a seed module based on an adjusted PPI network. To be specific, let a biological network be *G* and let the set of known disease proteins be *S*_0_, the IDMCSS performs seven main steps to detect a disease module. First, we initialize the disease protein set *S* to be the set of known disease proteins *S*_0_, and let the candidate disease protein set *C* be empty. Then, we select all the neighbors of known disease proteins, i.e., *NS* = (*b*_1_, …, *b*_*α*_), based on the current network *G*, where *b*_*i*_ (*i* = 1, …, *α*) is a neighbor of a certain node in *S*. Third, the structure of the current network is locally changed into a new network, *G*_*new*_, by the suggested network adjustment strategy, which focuses on removing the potential incorrect links and adding the potential missing links around the nodes in *S*. Fourth, the neighbors of the nodes in *S*, i.e., *NS*, are updated according to the adjusted network *G*_*new*_. Fifth, we select the protein *b* from *NS* which is most likely to be a disease protein by the suggested similarity, and add the node *b* into the set *S* and the candidate disease protein set *C*. The above the second to the fifth steps are repeated until a certain disease-related information (gene ontology, differential expression genes, pathways) is not significantly enriched in the set *C*, where the significance estimation used in Wen et al. (2013) is adopted here for enrichment analysis. Sixth, the subnetwork *G*_*s*_ is extracted from the adjusted network *G*_*new*_, where the node set of the subnetwork is *S*. Note that, *G*_*s*_ may be disconnected. Finally, the connected network with the largest number of nodes in *G*_*s*_ is selected as a disease module, denoted as *G*_*cs*_. Algorithm 1 presents the pseudo code of the framework of IDMCSS.

Algorithm 1 Framework of the IDMCS.

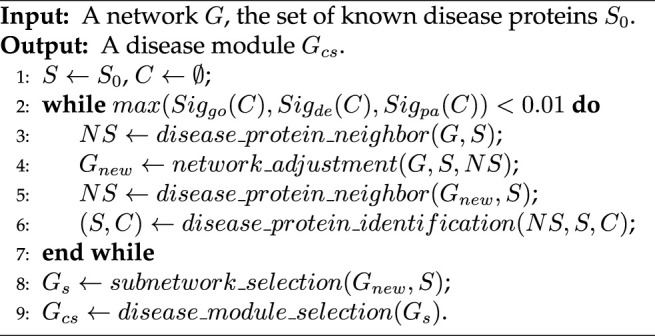

### 3.2 Network Adjustment Strategy

For the network *G* = (*V*, *E*) and the disease protein set *S*, the IDMCSS starts to locally change the network structure of the original network *G* around the nodes in *S*, in order to discard several potential incorrect links and retrieve several missing links in *G*. To this end, a network adjustment strategy is developed to focus on removing several potential incorrect links associated to the nodes in *S* and adding potential missing links between a node *S* and its neighbors. Algorithm 2 details the procedure of network adjustment strategy, which is performed as follows.

Algorithm 2 Network-adjustment (*G, S, NS*).

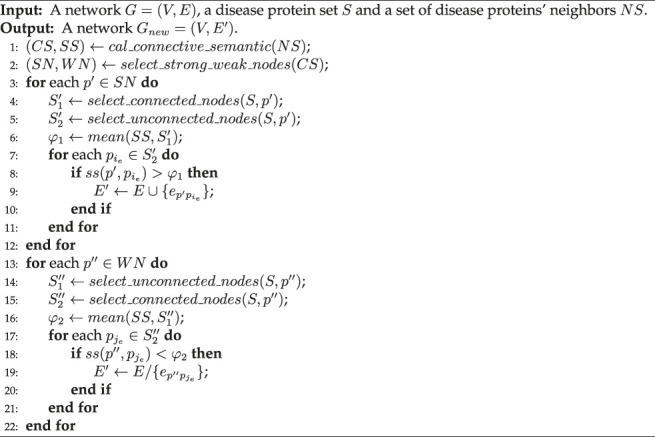

First, we calculate both the connective similarity and the semantic similarity between each protein in *NS* and the diseases proteins in *S* = (*p*_1_, …, *p*_*n*_). For a node *b* ∈ *NS*, it is supposed that the node *b* has the degree *k* and connects to *k*_*s*_ nodes in *S*. The connective similarity between node *b* and the nodes in *S* is calculated by a hypergeometric test as Eq. 1., which represents how closely protein *b* connects to disease proteins in *S* (Susan Dina et al., 2015).

$$cs\left(b,S\right)=1-\overset{k}{\underset{t=k_{s}}{\sum }}\frac{C_{n}^{t}C_{N-n}^{k-t}}{C_{N}^{k}},$$ (1)

where *n* is the number of nodes in *S*, and *N* is the number of nodes in *G*.

Then, we can calculate the semantic similarity between protein *b* and disease proteins *S*. Assume that the set $T=\left\{t_{i}|i=1,\ldots ,M\right\}$ consists of all of the terms annotating *N* proteins in network *G*.

$$ss\left(b,S\right)=\overset{n}{\underset{i=1}{\sum }}\frac{\sum_{t_{i}\in \left(A_{b}\cap A_{p_{i}}\right)}I\left(t_{i}\right)}{I_{max}\left(S\right)},$$ (2)

where $A_{b}=\left\{t_{x_{k}}|k=1,\ldots ,m\right\}$ and $A_{p_{i}}=\left\{t_{y_{j}}|j=1,\ldots ,m^{\prime }\right\}$ are the sets of terms used to annotate the proteins *b* and *p*_*i*_, and t_*_ represents a term in **T**. *I* (*t*_*i*_) =−*log* [*pro* (*t*_*i*_)] is the information of the term *t*_*i*_, where *pro* (*t*_*i*_) denotes the probability of the presence of the term *t*_*i*_ and its descendants in the term set **T**. The information of protein *p* is $I\left(p\right)=\sum_{k=1}^{m}I\left(t_{x_{k}}\right)$ . *I*_*max*_(*S*) = *max*[*I* (*p*_1_), …, *I* (*p*_*n*_)] denotes the largest value of the information of proteins in *S*.

Second, the strong-linked nodes (SN) and the weak-linked nodes (WN) are selected from *NS*, denoting proteins in *NS* closely and weakly related with disease proteins in *S*, where a strong-linked node is defined as the protein having a connective similarity with *S* larger than 0.99, and a weak-linked node is defined as the protein when it has a connective similarity with *S* smaller than the average value in *NS*. Note that, the connective similarity ranges from 0 to 1, and the average value of connective similarity is always smaller than 0.99. Thus, there is no intersection between the strong-linked nodes (SN) and the weak-linked nodes (WN). Third, the network *G* is changed to *G*_*new*_ by adding or removing several links associated with the strong-linked or weak-linked nodes, according to the suggested network adjustment strategy. The network adjustment strategy includes two key operators, i.e., adding and removing links, which are designed as follows.

1) Adding link operator: For a strong-linked node *p*′ ∈ *SN*, we check whether a link needs to be added between *p*′ and the node in *S* which is not connected with *p*′ in the current network. Let $S_{1}^{\prime }=\left\{p_{i_{1}},\ldots ,p_{i_{r}}\right\}\subseteq S$ and $S_{2}^{\prime }=S/S_{1}^{\prime }$ be the two sets of nodes which are connected and not connected to node *p*′. For each node $p_{i_{e}}\in S_{2}^{\prime }$ , a link between node *p*′ and node $p_{i_{e}}$ is added into the current network when $ss\left(p^{\prime },p_{i_{e}}\right)>\varphi_{1}$ . This means that a link is added if the semantic similarity $ss\left(p^{\prime },p_{i_{e}}\right)$ between *p*′ and node $p_{i_{e}}$ is larger than *φ*_1_, where *φ*_1_ is the mean semantic similarity between *p*′ and each node in $S_{1}^{\prime }$ .

**FIGURE 1:**
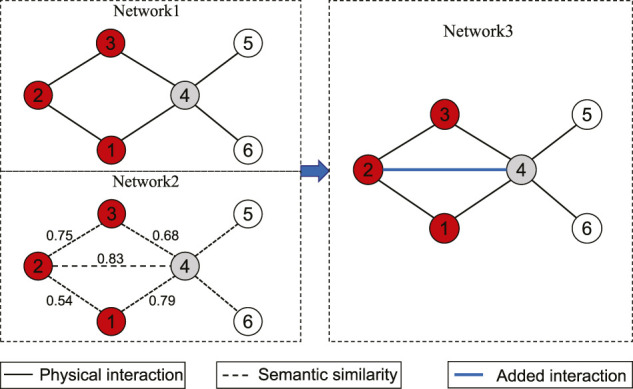
An illustrative example of the suggested adding link operator. Network 1 is the original network, where the red nodes denote disease proteins **1**, **2** and **3** in *S*, and the gray node **4** represents a neighbor of nodes in *S*, and node **4** is a strong-linked node; Network 2 represents the semantic similarity network, where the marked edge weights are the semantic similarity. Network 3 represents the adjusted network, where a link is added between nodes **2** and **4**.

Figure 1 presents an example to show how the suggested adding link operator works. As shown in this figure, the set of disease proteins *S* contains three nodes **1**, **2** and **3**, and *NS* = (**4**). For node **4**, $S_{1}^{\prime }=\left\{1,3\right\}$ includes the nodes in *S* which are connected with **4**, and $S_{2}^{\prime }=\left\{2\right\}$ contains node **2** which is not connected with node **4**. Node **4** is a strong-linked node in *NS*, since the connective similarity between node **4** and *S* = (1, 2, 3) is 0.9964 according to Eq. 1, which is larger than the threshold 0.99. Further, the link between node **2** and node **4** is added, since the semantic similarity between them is 0.83 which is larger than the threshold $\varphi_{1}=\frac{0.68+0.79}{2}$ .

2) Removing link operator: For a weak-linked node *p* ^″^ ∈ *WN*, the network adjustment strategy checks whether some links deserve to be removed to ensure that the weak-linked node *p* ^″^ is not connected to any node in *S*. Let $S_{1}^{\prime \prime }=\left\{p_{j_{1}},\ldots ,p_{j_{s}}\right\}\subseteq S$ and $S_{2}^{\prime \prime }=S/S_{1}^{\prime \prime }$ be the two sets of nodes which are not connected and connected to node *p* ^″^. For each node $p_{j_{e}}\in S_{2}^{\prime \prime }$ , a link between *p* ^″^ and $p_{j_{e}}$ is removed when the semantic similarity between *p* ^″^ and $p_{j_{e}}$ is smaller than *φ*_2_, where *φ*_2_ denotes the mean semantic similarity between node *p* ^″^ and each node in $S_{1}^{\prime \prime }$ .

**FIGURE 2:**
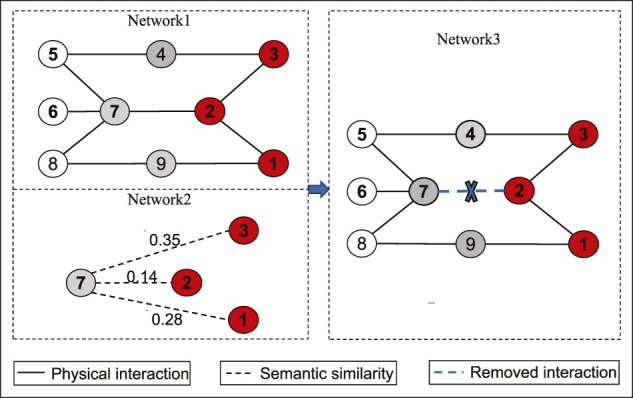
An illustrative example of the suggested removing link operator. Network 1 is the original network, where the red nodes denote disease proteins **1**, **2** and **3** in *S*, and the gray nodes **4**, **7**, and **9** represent the neighbors of nodes in *S*, i.e., *NS* = (4, 7, 9); Node **7** is a weak-linked node in *NS*. Network 2 represents the values of the semantic similarity between **7** and each node in *S*. Network 3 represents the adjusted network, where the link between nodes **7** and **2** is removed.

Figure 2 presents an illustrative example of the removing link operator. In this example, *S* = (1, 2, 3) represents the set of disease proteins and *NS* = (4, 7, 9) consists of all neighbors of nodes in *S*. For node **7**, there are two nodes 1 and 3 which are not connected with it ( $S_{1}^{\prime \prime }=\left\{1,3\right\}$ ), and one node 2 which is connected with it ( $S_{2}^{\prime \prime }=\left\{2\right\}$ ). By simple calculation, we can obtain that the connective similarity between node **7** and set *S* is 0.9964 and the average connective similarity of the nodes in *NS* is 0.9984. Since the connective similarity is smaller than the average value, the node **7** is weak-linked. Hence, we need to remove the link between nodes **7** and **2** from the network, due to the fact that the threshold $\frac{0.35+0.28}{2}$ is larger than the semantic similarity between nodes **7** and **2** in $S_{2}^{\prime \prime }$ (i.e., 0.14).

### 3.3 The Similarity Between a Protein and Disease Proteins

In the IDMCSS, the protein having the largest similarity with the nodes in *S* is selected as a disease protein, where the similarity is measured based on both connective similarity and semantic similarity. Specifically, considering a protein *p* and a set of disease proteins *S* = (*p*_1_, …, *p*_*t*_), the similarity between the protein *p* and the set of disease proteins *S*, denoted as *sv* (*p*, *S*), is the normalization of the sum of the connective similarity and the semantic similarity, which is defined as Eq. 3.

$$sv\left(p,S\right)=\frac{cs\left(p,S\right)+ss\left(p,S\right)}{2},$$ (3)

where *cs* (*p*, *S*) represents the connective similarity between *p* and *S*, and *ss* (*p*, *S*) represents the semantic similarity between *p* and *S*.

### 3.4 Complexity Analysis

Here, an upper bound of the time complexity of the IDMCSS is presented. As described above, the main complexity of IDMCSS lies in the following five steps: 1) the identification of *NS*, 2) the network adjustment, 3) the selection of disease protein, 4) extracting the subnetwork *G*_*s*_ from the adjusted network, 5) selecting a disease module *G*_*cs*_. Note that, the first three steps are in a while loop.

The complexity for the identification of *NS* is *O* (*d*_*max*_ × *n*), where |*S*| = *n*, the largest degree of nodes in *S* is *d*_*max*_. Suppose the number of nodes in *NS* is *n*′, a complexity of *O* (4 × *n*′ + *n*′^2^) is needed for the network adjustment, since the complexity for calculating connective and semantic similarity as well as selecting strong and weak nodes is *O* (4 × *n*′), and the maximum complexity for adding and removing links is *O* (*n*′^2^). The maximum complexity for the selection of disease protein is *O* (*n*′). The first three steps holds a time complexity of *O* (*d*_*max*_ × *n* + *n*′^2^), since *O* (*d*_*max*_ × *n* + *n*′^2^) ≈ *O* (*d*_*max*_ × *n* + 4 × *n*′ + *n*′^2^ + *n*′). After the iteration of *maxgen* times, it needs a complexity of *O* ((*d*_*max*_ × *n* + *n*′^2^) × *maxgen*) for identifying the disease proteins. The fourth step needs a time complexity of *O*(*M*) to extract the subnetwork *G*_*s*_ from the adjusted network *G*_*new*_, where *M* is the number of links in *G*_*new*_. Finally, it holds a time complexity of *O* (*M*′) to select a disease module, where *M*′ is the number of links in *G*_*s*_. Therefore, the IDMCSS holds a computational complexity of *O* (*d*_*max*_ × *n* × *maxgen* + *n*′^2^ × *maxgen* + *M*), since *O* ((*d*_*max*_ ×*n* + *n*′^2^) ×*maxgen* + *M* + *M*′) ≈ *O* (*d*_*max*_ ×*n*×*maxgen* + *n*′^2^ ×*maxgen* + *M*).

## 4 Experimental Results

In this section, we first analyze the module of asthma obtained by the proposed IDMCSS, and then compare the performance of the IDMCSS with that of four existing algorithms for disease module detection.

### 4.1 Datasets

The IDMCSS performs the detection of asthma-related modules based on the protein-protein interaction network. The stopping criterion of the algorithm is set according to the information of gene ontology, differential expression genes and pathways which are related to the asthma. Specifically, the protein-protein interactions, microarray expression data, asthma-related genes and pathways are presented as follows.

First, the protein-protein interaction network is obtained by considering seven kinds of physical interactions simultaneously, which yields a network having 13, 460 proteins and 141, 296 physical interactions. The seven physical interactions considered here are regulatory interactions (Matys et al., 2003), biophysical interactions Aranda et al. (2009), Ceol et al. (2007), literature curated interactions Prasad et al. (2009), metabolic enzyme-coupled interactions Lee et al. (2008), protein complexes Ruepp and et al. (2010), kinase network Hornbeck and et al. (2012) and signaling interactions Vinayagam and et al. (2011) in human interactome. From the gene ontology annotation database (GOA) Huntley and et al. (2015), we extract 19, 707 genes annotated with GO terms and hence the obtained network consists of 12, 562 proteins and 130, 390 physical interactions.

Next, we adopt nine asthma-related microarray expression data sets consisting of the gene expression values for the differential expression analysis. The nine data sets are GSE470, GSE2125, GSE3004, GSE4302, GSE16032, GSE31773, GSE35571, GSE41649 and GSE43696, which can be available from the NCBI Gene Expression Omnibus database (GEO)^1^. It is worth noting that we use 107 known asthma-related genes in the protein-protein interaction network for experimental analysis in this paper, which are compiled from pervious literature Vercelli (2008) and several datasets^2^. In addition, 23 asthma-related pathways collected from the literature (Song and Lee, 2013; Sharma et al., 2012) are used in this paper (Supplementary Appendix S1).

### 4.2 Identification of Disease Modules

We use the IDMCSS to identify disease modules based on an adjusted network, where the final disease module of asthma is achieved by running the proposed IDMCSS 217 iterations. The reason for the iterations for 217 times is that “differential expression genes” is not significantly enriched in current disease proteins earlier than “GO annotation information” and “pathway information”, and the enrichment of the differential expression genes included in the disease proteins is smaller than 0.05 when the algorithm iterates 218 times.

For the disease module of asthma obtained by the suggested IDMCSS, it consists of 279 nodes and 2,819 links. Among the 279 nodes, 62 nodes are known asthma-related proteins and the other 217 nodes are newly discovered relating to asthma-related proteins. In the 2,819 links found in the disease module, 489 links are newly added and 19 links are removed from the original network by the proposed IDMCSS. It is worth noting that some known disease proteins associated with asthma are not included in the obtained disease module of asthma and hence they may be included in other connected subgraphs.

Finally, we take a close look at the closeness of the obtained disease module. We here use the ratio of the number of inner-links to that of external-links as the closeness of the disease module. The module has 2,819 inner-links and 47,657 external-links, and thus the closeness of the disease module is 0.0592. This confirms that the disease module is not a locally dense community as stated by Susan Dina et al. (2015). It can also be found that the obtained disease module has statistically larger closeness than the subnetworks randomly selected from the adjusted protein-protein interaction network according to the Student’s t-test.

### 4.3 Asthma-Related Pathways and Genes in the Disease Module

In this subsection, we analyze the asthma-related pathways and genes in the disease module. To this end, from 304 human pathways in the Biocarta database given in Supplementary Appendix S2, we extract the 72 candidate pathways which has at least half of genes in the disease module obtained by the algorithm. It can be found that the 72 pathways are possible asthma-related pathways as shown in Supplementary Appendix S3, since they are statistically significantly enriched in the disease module. Among the 72 pathways, two are included in the 23 known asthma-related pathways and the rest 70 are the newly asthma-related pathways predicted by the algorithm. For the 70 pathways, five pathways, “h-il7Pathway”, “h-pkcPathway”, “h-melanocytepathway”, “h-ngfPathway”, and “h-trkaPathway”, are considered to be associated with asthma in previous literature (Kelly and et al., 2009; Hou and et al., 2017; Raap et al., 2003; Abram, 2008).

Next, we will predict several targets of glucocorticoid based on the disease module of asthma, since they are an effective anti-inflammatory drug for asthma. The genes will be considered as the targets of glucocorticoid in asthma if they are differentially expressed between asthmatic fibroblasts untreated and asthmatic fibroblast cells treated with glucocorticoid, but not between normal untreated fibroblast cells and normal fibroblasts treated with glucocorticoid. For this reason, in this paper the 12 genes, *acvrl1*, *ar*, *cdk1*, *ctgf*, *ddit3*, *icam1*, *jak1*, *rora*, *smad1*, *snca*, *tgfb2*, and *tlr4*, are considered to be targets of glucocorticoid. To verify the effectiveness of the targets, we use the enrichment analysis of the differential expression genes before and after the treatment of glucocorticoid. For 217 expanded proteins, 23 and 17 expanded proteins are differentially expressed in normal and asthmatic samples, respectively. As for the 62 known asthma-related proteins, 10 and 8 known asthma-related proteins are differentially expressed in normal and asthmatic samples, respectively. Based on the Fisher’s exact test, in normal and asthmatic samples the expanded proteins have the enrichment of differential expression genes 6.0324 × 10^−4^ and 2.70, ×, 10^−3^, and the known asthma-related proteins have the enrichment of differential expression genes 4.32 × 10^−2^ and 4.30, ×, 10^−2^. This means that the expanded proteins has significantly higher enrichment of differential expression genes than the known asthma-related proteins. Thus, we can conclude that the algorithm can provide effective targets for therapeutic intervention.

### 4.4 Robustness of IDMCSS

**FIGURE 3:**
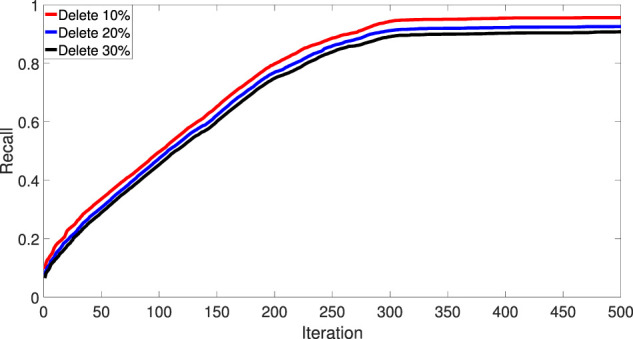
The recall rate of disease module.

To show the robustness of IDMCSS, Figure 3 gives the recall rate of the disease module when 10, 20, and 30*%* of the known asthma disease genes are randomly deleted, averaging over 30 times experiments (Warren et al., 2002). It can be found that the removal of the known disease genes has little influence on the performance of the suggested IDMCSS, and it always detect similar disease modules in the 217 iterations. Hence, we can conclude that the suggested IDMCSS shows a good robustness in detecting disease modules of asthma.

### 4.5 Performance Comparison

The IDMCSS is compared to four state-of-the-art disease module identification approaches, including a network structure change-based algorithm (CTSS) (Liu et al., 2015) and three traditional approaches without changing network structures (DIAMOnD Susan Dina et al. (2015), RWR Sebastian et al. (2008) and HRSS Wu et al. (2013)), where DIAMOnD and RWR are connective-based algorithms and HRSS is a semantic-based algorithm. Specifically, CTSS identifies disease genes by adding weak interactions between unconnected genes in the existing network based on the semantic similarity between them. The DIAMOnD algorithm is a seed-expanding method which identifies a disease module around a set of known disease proteins in the PPI network. RWR uses random walk analysis, which is a global network distance measure, to measure similarities among proteins in the PPI network. HRRS ranks all nodes by calculating the relative specificity similarity of each node in the network to known disease nodes, where the relative specificity similarity is calculated by taking the global position of relevant gene ontology terms into account. For the above comparison algorithms, the best parameters recommended in their original references are adopted.

**FIGURE 4:**
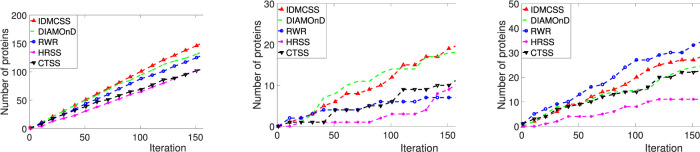
Performance of IDMCSS, CTSS, DIAMOnD, RWR and HRSS on the asthma dataset. GO annotations: the number of asthma-related GO annotations enriched in the disease module; DifferExpre: the number of differential expressed genes in the disease module; Pathways: the number of asthma-related pathways enriched in the disease module.

Figure 4 presents performance (the number of proteins annotated by asthma-related GO terms, the number of differential expression genes, and the number of proteins in asthma-related pathways) obtained by five approaches on the asthma dataset. To be specific, the left one in Figure 4 draws the number of proteins which are significantly annotated by 940 asthma-related GO terms for different iterations, where the 940 asthma-related GO terms are those enriched in the 107 known asthma proteins (Supplementary Appendix S4). From the figure, it can be found that IDMCSS achieves the largest number of proteins annotated by asthma-related GO terms.

The middle one in Figure 4 plots the number of differential expression genes included in the disease module achieved by IDMCSS and those by four compared algorithms when the iteration ranges from 1 to 217. As can be seen from the figure, the algorithm IDMCSS gains the largest number of differential expression genes when the iteration is larger than 111. The main reason may be attributed to the fact that by enhancing the structure of PPI, it becomes relatively easy to detect the differential expression genes, thus the IDMCSS can achieve a competitive performance in detecting disease modules. The right one in Figure 4 presents the number of proteins which belong to the 23 known asthma-related pathways. It is found that the IDMCSS is slightly worse than RWR, but it is better than other algorithms. The main reason for the phenomenon is that the proteins linked by physical interactions tend to collaborate with each other in the same pathway (Venkatesan et al., 2008. The proteins obtained by RWR are always the known disease proteins’ neighbors which are connected to the known disease proteins by physical interactions in the PPI network, while those obtained by IDMCSS may be the nodes which are not linked with the known disease proteins. Therefore, we can conclude that the IDMCSS is a competitive disease module detection algorithm in terms of detection quality.

## 5 Conclusion and Future Work

^3^In this paper, we have developed a disease module identification method IDMCSS by modifying the existing PPI networks. In the suggested IDMCSS, some potential interactions are added in the existing PPI network and some incorrect interactions are removed based on the connective and semantic similarities between the given disease proteins and their neighboring proteins. The basic idea of modifying the existing PPI network is that the incorrect links and the missing links are in the original PPI network, and we want to eliminate interference of the incorrect links and missing links for detecting disease module. However, due to the lack of the knowledge about the accurate protein-protein interactions, it is hard to analyze the validity of the modified PPI network, which may be verified in the future. The protein having the best connective and semantic similarities in the neighborhood of known disease proteins is extended into the set of disease proteins on the adjusted PPI network step by step until a stopping criterion is reached. Further, the connected subgraphs which include the disease proteins, as well as the interactions between them, are extracted from the adjusted network. Finally, the connected subgraph which contains the largest number of disease proteins is selected as a disease module.

We have performed a series of experiments on a particular disease, i.e., asthma to show the effectiveness of the IDMCSS. First, the disease module detected by the IDMCSS was not a dense community which is in accordance with traditionary discovery, and it was also significantly different from the random subgraphs. Then, several pathways and genes discovered in the disease module have been verified to be related to asthma. Further, IDMCSS has little sensitivity to the number of known disease proteins. Finally, IDMCSS was superior to state-of-the-art approaches for disease module identification, since the disease module achieved by IDMCSS includes more proteins which are enriched in asthma-related GO terms, pathways and differential expression genes than those achieved by other approaches. From the above, the experiments have extensively demonstrated the superiority of IDMCSS in disease module identification.

In this work, we have locally adjusted the network structure by the suggested network adjustment strategy to deal with the PPI network which suffers from both high false positive and false negative rates. The IDMCSS performs based on the assumption that the detection results will become better if the PPI network becomes more perfect. Future attention can be given to combing connective information with other kinds of information, such as pathway information and phenotypic similarity information, to further improve the IDMCSS.

## Data Availability

Publicly available datasets were analyzed in this study. This data can be found here: In this paper, we adopt nine asthma-related microarray expression data sets consisting of the gene expression 252 values for the differential expression analysis. The nine data sets are GSE470, GSE2125, GSE3004, GSE4302, 253 GSE16032, GSE31773, GSE35571, GSE41649 and GSE43696, which can be available from the NCBI Gene Expression Omnibus database (GEO) http://www.ncbi.nlm.nih.gov/geo/.

## Footnotes

1
http://www.ncbi.nlm.nih.gov/geo/

2
http://gene2mesh.ncibi.org

3 This paper is an extended version of a paper of our published in the 14th International Conference on Bio-inspired Computing: Theories and Applications (BIC-TA 2019).
